# Prevalence of active trachoma and associated factors among school age children in Debre Tabor Town, Northwest Ethiopia, 2019: a community based cross-sectional study

**DOI:** 10.1186/s13052-022-01258-x

**Published:** 2022-05-03

**Authors:** Alebachew Shimelash, Mekuriaw Alemayehu, Henok Dagne, Getenet Mihiretie, Yonas Lamore, Eniyew Tegegne, Lake Kumlachew

**Affiliations:** 1 Department of Environmental Health, College of Health Sciences, Debre Markos University, Debre Markos, Ethiopia; 2 Institutes of Public Health, College of Medicine and Health Sciences, University of Gondar, Gondar, Ethiopia; 3 Department of Public Health, College of Health Science, Debre Tabor University, Debre Tabor, Ethiopia

**Keywords:** School-age children, Active trachoma, Risk factor, Debre Tabor Town, Ethiopia

## Abstract

**Background:**

Trachoma is an infectious eye disease caused by *Chlamydial trachomatis*. It is a major health problem in poor nations, notably in Sub-Saharan Africa. Despite the severity of the problem, there was a scarcity of data on trachoma prevalence and associated factors among school-aged children in Debre Tabor town following SAFE and MDA.

**Objectives:**

The goal of this study was to determine the prevalence of active trachoma and its associated factors among school-aged children in Debre Tabor, Northwest Ethiopia, in 2019.

**Methods:**

A community-based cross-sectional study was used among school-aged children. Structured interview questionnaires, an observational checklist, and a physical examination were used to collect data from study participants who were chosen using a systematic random sampling procedure. IBM SPSS 20 was used to enter data, which was then transferred to IBM SPSS 20 for bivariate and multivariable logistic regression analysis.

**Result:**

A total of 394 children aged 5–15 had been screened and took part in the study, with 9.9% (95% CI: 6.9, 12.7) testing positive for active trachoma. Having an unimproved larine type (AOR = 5.18; 95%CI: 1.96, 13.69), improper solid waste disposal (AOR = 3.026; 95%CI: 1.17, 7.8), family size greater than four (AOR = 3.4; 95%CI: 1.22, 9.49), not using soap for face washing (AOR = 4.48; 95%CI: 1.46, 13.72) and an unclean face of the child during examination (AOR = 23.93; 95%CI: 8.25, 69.38) were found to be significant predictors of active trachoma.

**Conclusion:**

Active trachoma among school-age children was high compared to the WHO’s definition of trachoma as a public health problem. A family size of four, poor solid waste management, an unimproved type of latrine, an unclean child's face, and not using soap when washing one's face were all significant predictors of active trachoma. Promotion of behavioral determinants through health education programs like keeping facial cleanliness by washing their child’s face with soap, managing solid waste properly, and installing improved latrines to reduce active trachoma needs to be in place.

## Background

Chlamydia trachomatis is an infectious eye disease caused by *Chlamydia trachomatis, an* obligate intracellular bacterium. On some occasions, it begins as follicular conjunctivitis, with superficial keratitis and corneal vascularization, and advances to conjunctiva scarring and lid distortion. It is the leading cause of blindness worldwide, and it is spread by eye-to-eye contact, transmission on fingers, fomites, coughing and sneezing, and eye-seeking flies[1, 2].

Approximately 1.3 million people are blind from trachoma, and probably 1.8 million have low vision. Trachoma is endemic in more than 50 countries, predominantly in sub-Saharan Africa, including the Middle East and Asia[3, 4]. According to the WHO weekly epidemiologic record, there were 157.7 million individuals living in districts where active trachoma was a public health hazard in 2018, with 88 percent of them in Africa and half of them (69,802,693) in Ethiopia [5].

The nationwide prevalence of active trachoma (either TF or TI) among children aged 1 to 9, was 40.14 percent in 2007. The prevalence varied significantly by location; the highest prevalence was found in Amhara (62.6%) [6], where Debre Tabor is found. The World Health Organization (WHO) and other concerned bodies have targeted trachoma for elimination by 2020 by implementing the so-called SAFE strategy (surgery for in-turned eyelashes, antibiotics to clear infection, and facial cleanliness and environmental improvement to reduce infection transmission). The global trachoma program has brought considerable success with the SAFE strategy, as 9 formerly endemic countries have recently been eliminated to a level of no more public importance[7, 8].

Although they vary among settings, factors like increased family size and the number of children in the household that create crowded living conditions increase the probability of transmission of active trachoma[9]. Childhood hygiene behaviors such as ocular and nasal secretions and unclean faces attract flies and pave the way for transmission[10]. Likewise, water scarcity also promotes the transmission, which in turn compromises hygienic practices, like face washing. Limited access to latrines increases fecal contamination of the environment, which favors Fly breading is also a mechanical vector for trachoma transmission [11–14].

Despite the SAFE strategy, which included an annual Mass Drug Administration (MDA) with azithromycin, trachoma remained hyper-endemic in Amhara National Region State, including Debre Tabor town [15, 16]. Understanding the infection and its distribution across different study populations would be beneficial for program planning in hyper-endemic areas. As a result, the goal of this study is to fill in the gaps in knowledge on active trachoma burden and its determinants following SAFE and MDA in the elimination roadmap of neglected tropical disease in Ethiopia.

## Methods

### Study design and settings

A community-based cross-sectional study was conducted among school-age children from April 1–30, 2019. The study area, Debre Tabor town, is located 97 km from Bahir Dar, the capital city of Amhara National Regional State, and 667 km from Addis Ababa, the capital of Ethiopia. The town has an estimated population of 60, 563 people and is subdivided into 6 kebeles.

### Sample size and sampling procedures

The sample size was calculated by using EPI INFO version 7.2.0.1 based on the associated factors of active trachoma among school-age children, considering the following assumption: outcome unexposed to outcome exposed = 1, power: 80%, level of confidence: 95%, type I error: 5%, margin of error: 1.5%, design effect: 1.5%, and non-response rate: 10%. The final sample size was 401. Initially, 3 kebele were selected using simple random sampling by the lottery method. The final sample size was proportionally allocated to each selected kebele, and systematic random sampling was employed to draw the study participants. If more than one school-age child was found in the same household, one child was selected randomly using the lottery method.

### Data collection tools and procedures

Face-to-face interviews, observations, and physical examinations were used to collect data.Detailed information regarding socio-demographic factors, behavioral factors, and psychosocial factors were also included in the structured questionnaire. Four senior Integrated Eye Care Workers (IECWs) were involved in grading trachoma by assessing diagnostic signs using 2.5X loupes as recommended elsewhere[17]. IECWs swept their hands using alcohol after each examination. Eight urban health extension professionals collected the socioeconomic characteristics, environmental and behavioral factors. They have worked in health centers and were properly trained, experienced, and certified by the carter center Ethiopia.

### Data quality control

The data collection tool was first designed in English and then translated into the local language, Amharic, and then back to English to check its consistency. Secondly, training was provided for four data collectors and two supervisors for two days to familiarize them with the data collection procedures. Finally, a pre-test was conducted on 5% (*N* = 20) of the total sample size. Based on the pre-test analysis, unclear questions were edited and modified. The collected data was evaluated by supervisors and investigators for completeness, accuracy, and clarity.

### Operational definition

**Active trachoma**: the presence of Trachomatous inflammation, follicles/TF (the appearance of five or more follicles with a diameter of greater than 0.5 mm in the central part of the upper tarsal conjunctiva) and/or Trachomatous inflammation intense/TI (pronounced inflammatory thickening of the tarsal conjunctiva that obscures more than half of the normal deep tarsal vessels) on one or both eyes[18].

**Facial cleanliness:** The absence of nasal and ocular discharge on the face[18].

**Improved latrine:** a form of latrine which hygienically separate excreta from human contact[19].

**Proper solid waste management:** solid waste disposed at the household level by using sacks/disposal pit and composting at the household level[20].

**Proper liquid waste management:** liquid waste disposed of at the household level by using one of the individual containment systems (septic tank, soak pit, cesspool or seepage pit).

**Improved water sources:** water from the following sources; piped household water connection, public taps or standpipes, tube wells or boreholes, protected dug wells, protected springs, and rainwater collection[21].

**School-age children:** Children between 5 and 15 years of age who may or may not be enrolled in school[22].

**Kebele:** the smallest administrative unit in Ethiopia.

### Data processing and analysis

The data was coded, cross-checked, and entered into EPI INFO Version 7.2.0.1 and exported to SPSS 20 for analysis. Frequency, percentage, mean, and standard deviation have been computed to describe the prevalence of active trachoma. Variables with *p* values of ≤ 0.2 in the bivariate analysis were fitted to multivariable logistic regression.

The association between the different variables and the presence of active trachoma was measured using odds ratios with 95% CIs of crude odds ratios (CORs) and adjusted odds ratios (AORs). Statistical significance was declared when the *p*-value was ≤ 0.05.

## Results

### Socio-demographic characteristics

Three hundred ninety-four school-age children were examined out of four hundred one (401), with an overall response rate of 98.25%. More than half of the study participants (82.2%) were female. The majority of study subjects (80.7%) lived in households headed by a male. Majority of guardians (62.9%), were married less than or equal to 18 years old. Children’s educational levels showed that 24.6% had not attended the school, 54.8% were in grades 1^st^ –4^th^ and 20.6% were in grades 5th–8^th.^ The average age of school-age children was 8.99 (standard deviation) 2.68 years (Table 1).

**Table 1:** Socio-demographic characteristics among school-age children at Debre Tabor town, Northwest Ethiopia, May 2019 (*n* = 394)

Variables	Category	Frequency(*n* = 394)	Percent
Sex of respondent	Female	324	82.2
	Male	70	17.8
Age of guardians	18–29 years	92	23.3
	30–44 years	247	62.7
	≥ 45yrs	55	14
Years of living in the family	1–4 yrs	66	16.8
	≥ 5 yrs	328	83.2
Religion	Orthodox	378	95.9
	Muslim	15	3.8
	Catholic	1	0.3
Respondent role in the family	Father	45	11.4
	Mother	308	78.2
	Son	11	2.8
	Daughter	30	7.6
Educational status of the care giver	Unable to read and write	95	24.1
	Primary school	119	30.2
	Secondary and higher	180	45.7
Household head educational status(*n* = 318)	Unable to read and write	45	14.1
	Primary school	88	27.7
	Secondary and higher	185	58.2
Marriage age of mother/guardian	≤ 18 years	248	62.9
	> 18 years	146	37.1
Occupational status of the mother	Daily laborer	53	13.5
	Governmental employee	78	19.8
	Private employee	36	9.1
	House wife	170	43.1
	Merchant	44	11.2
	Others	13	3.3
Household head occupational status(*n* = 318)	Daily laborer	39	12.3
	Governmental employee	146	46
	Private employee	50	15.7
	Merchant	67	21
	Others	16	5
Monthly income of the family	200–1000 birr	117	29.7
	1001–2750	80	20.3
	2751–5000	120	30.5
	Greater than 5001	77	19.5
Family size	≥ 4members	209	53
	< 5members	185	47
Under 15 years of children	1 child	155	39.3
	≥ 2 children	239	60.7
Sex of child	Male	221	56.1
	Female	173	43.9
Age of child	5–9 years	227	57.6
	10–15 years	167	42.4
School grade	Currently not attended the school	97	24.6
	Grade 1–4	216	54.8
	Grade 5 -8yeras	81	20.6

### Environmental characteristics

The majority (73.9%) of children were living in households with properly disposed of solid waste at the household level. Almost all (98%) children were living in households with an improved source of water (Table 2).

**Table 2:** Frequency distribution of Environmental factors associated with the prevalence of active of trachoma at Debre Tabor town Administration, Northwest Ethiopia, May 2019 (*n* = 394)

Variables	Category	Frequency(*n* = 394)	Percent
SWM at HH	Improper	103	26.1
	Proper	291	73.9
LWM at HH	Improper	210	53.3
	Proper	184	46.7
Source of water	Improved water source	386	98
	Unimproved water source	8	2
Daily HH water consumption	20–40 L	286	72.6
	41–60 L	88	22.3
	≥ 61 L	20	5.1
Availability of latrine	Yes	376	95.4
	No	18	4.6
Latrine type (= 376)	improved	273	72.4
	Unimproved	105	27.6
House ownership	Owner	255	64.7
	Rental	139	35.3

*SWM* Solid Waste Management, *LWM* Liquid Waste Management, *HH* Household

### Behavioral characteristics of face washing

The majority (99.7%) of children wash their faces on a daily basis, with 190 (49.7%) using soap in the process. 35.8% of children wash their face at least once a day, 49.7% twice day and 14.8% three times and more. 88.8% of Children have a clean face (Table 3.).

**Table 3:** Frequency distribution of behavioral factors associated with the prevalence of trachoma at Debre Tabor town, Northwest Ethiopia, May 2019 (*n* = 394)

Variables	Category	Frequency (*n* = 394)	Percent
Ocular discharge	Yes	12	3
	No	382	97
Nasal discharge	Yes	44	11.2
	No	350	88.8
Facial cleanliness	Clean	349	88.6
	unclean	45	11.4
Utilization of fomites	Yes	110	27.9
	No	284	72.1
Utilization of soap	Yes	196	49.7
	No	198	50.3
Face washing habit	Never	1	0.3
	Some times	97	24.6
	Most of the time	162	41.1
	Always	134	34
Face washing frequency	Once a day	141	35.8
	Twice a day	196	49.7
	Three times and more	57	14.5

### Prevalence of active trachoma

The overall prevalence of active trachoma among school-age children was 9.9% (95% CI: 6.9–12.7%). The prevalence of TF and TI was 7.4% and 7.1% respectively.

### Factors associated with active trachoma

Bivariate analysis and multivariate analysis were computed to examine the association between active trachoma and socioeconomic variables, environmental factors, facial cleanliness, nasal and ocular discharge, utilization soap during face washing, and the knowledge of household head about trachoma. Those factors fulfilling the minimum criteria (P ≤ 0.2 of significant level) in bivariate regression analysis were fitted to multivariable logistic regression analysis.

Improper household solid waste management at household level (AOR = 3.02, 95% CI: 1.17–7.80), unimproved latrine (AOR = 5.18, 95% CI: 1.95–13.69), unclean face of the child (AOR = 23.93, 95% CI: 8.25–69.38), utilization of soap for face washing (AOR = 3.27, 95% CI: 1.19–8.94), and family size > 4 (AOR = 3.40, 95% CI: 1.22–9.49) were independent predictors of active trachoma(Table 4).

**Table 4:** Bivariate and multivariable analysis between variables and active trachoma among school-age children at Debre Tabor town, Northwest Ethiopia, 2019 (*N* = 394)

Variables	Category	Active trachoma		COR 95%CI	AOR 95%CI
		**Yes**	**No**		
SWM at HH level	Improper	20	83	3.45(1.76–6.77	3.02(1.17–7.80) *
	Proper	19	272	1	1
LWM at HH	Improper	28	182	2.42(1.17–5.01	1.34(0.37–4.88)
	Proper	11	173	1	1
Source of water	Unimproved	7	6	12.72(4.03 40.14)	6.52(0.58–72.99)
	Improved	32	349	1	1
Availability of latrine	No	5	14	3.58(1.21–10.55)	2.44(1.85–4.99)
	Yes	34	341	1	1
Latrine type(*n* = 376)	Unimproved	24	80	7.86(3.60–17.30)	5.18(1.95–13.69) **
	Improved	10	262	1	1
House property	Rental	18	121	1.66(0.85–3.23)	1.30(0.41–4.11)
	Owner	21	234	1	1
Nasal discharge	Yes	24	21	24.44(11.64–55.59)	0.29(0.05–1.61)
	No	15	334	1	1
Facial cleanliness	Unclean	24	22	24.21(11.14–52.62)	23.93(8.25–69.38)**
	clean	15	333	1	1
Utilization of fomites	Yes	17	98	2.00(1.03 -3.97)	2.56(0.87–7.50)
	No	22	257	1	1
Utilization of soap	No	30	168	3.71(1.71–8.04)	3.27(1.19–8.94) **
	Yes	9	187	1	1
Family size	> 4	23	162	1.71(0.87–3.35)	3.40(1.22–9.49) *
	≤ 4	16	193	1	1
School level of the child	Not attended	15	82	1.93(0.75–5.00)	2.65(0.55–12.01
	Grade 1–4	17	199	0.90(0.36–2.27)	1.21(0.29–5.09)
	Grade 5–8	7	74	1	1
Mother/guardian educational level	Unable to read and write	17	78	4.69(1.94–11.30)	1.39(0.28–6.76)
	Primary school	14	105	2.87(1.16–7.06)	1.89(0.46–7.69)
	Secondary and higher	8	172	1	1
Heard about trachoma	No	11	44	2.78(1.29–5.97)	0.93(0.22–3.88)
	Yes	28	311	1	1

*SWM* Solid Waste Management, *LWM* Liquid Waste Management, *HH* Household, *COD* Crude Odds Ratio, *AOR* Adjusted Odds Ratio

NB: The model adequately fit the data at a *P*-value = 0.261(Hosmer Lemeshow goodness Chi-square of 7.69), * significant at *p* value < 0.05, ** significant at *p* value < 0.01

## Discussion

The finding revealed that active trachoma was high, and is a significant public health problem among school-age children in Debre Tabor town compared to. The overall prevalence of active trachoma was 9.9% (95% CI: 6.9–12.7%). The prevalence of TF and TI were 7.4% (95%CI: 5.1–10.1) and 7.1% (95%CI: 4.6–9.6), respectively.

The prevalence of this study was comparable with results reported from isolated treatment-nave island communities of West Africa (14.7%)[23], Gambela (12.3%)[24], Gondar Zuria (12.1%)[25], and Leku town (11%)[26], These slight variations might be due to the fact that school age children who were attending schools could wash their face and get health education about personal hygiene and active trachoma at school [27], and also might be due to mass drug administration implementation in Debre Tabor town [28] which had reduced the level of active trachoma infection. But, the prevalence was higher compared to the studies in Brazil (3.4%)[29], Shanghai, China (5.2%)[30],Yunnan province (0.2%)[31], India (6.8%)[32], Mali (0.53%)[33], Pakistan (1.91%)[34], Harari region (1.3%) [35], Mali (6.2%), Niger(4.6%), and Nigeria (4.2%) [36], and Benishangul Gumuz Region (7,4%)[37]. High prevalence of active trachoma in this study area might be due to the immigration of people into the town fleeing from civil wars across different areas of the country[38]. Hundreds of thousands of people, mostly women and children, were forced from their homes and livelihoods as a result of the fighting in northern Ethiopia[39], which in turn, overburdens the capacity of water, sanitation, and hygiene facilities.

However, the prevalence in this study was lower than in studies in the Southern Nation Nationalities Peoples Region(> 10%) [40], Madda Walabu (22%) [41], Gonji Kolella (23%)[42], Zala district (36.7%)[43], Gazegibela (52.4%) [44], evaluation survey of active trachoma prevalence in Ethiopia (19.1%)[36], and Sokoto State (37%)[45]. This might be the current finding, and the previous studies mentioned above were not similar by the age of the study population. On the other hand, it might also be due to better access to safe water, improved sanitation and health facilities in areas where this study was conducted compared to the previous studies. In addition, Gazegibela district is repeatedly drought-affected and food-insecure, which is attributed to the high prevalence of trachoma[44].

Multivariable logistic regression analysis implies, the odds of having active trachoma among school-age children from households having greater than 4 family sizes was 3.4 times higher than those from households that had less than or equal to 4 family sizes (AOR = 3.4; 95% CI:1.22–9.49). This result was supported by research conducted in the Brazilian amazon [29], Harari Region [35] and Leku town [26]. This may be due to a lack of time to guardians to take care as the number of school-age children, and families are rising within the household. Besides, the infection rate and severity of trachoma are closely related to the overcrowded living conditions of the households [46, 47]. The probability of direct human contact, sharing sleeping places, towels, and clothing might be increased for ocular Chlamydia to spread[48].

The odds of having active trachoma among children who had unclean faces were 23. 93 times higher than those who had clean face (AOR = 23.93; 95% CI: 8.25–69.4). Similar findings were reported in Mali [33], Zala [49], and Ethiopia [50]. This might be due to the fact that an unclean face attracts eye- seeking flies that transmit trachoma mechanically. Children with unclean faces could be more likely to spread nasal and ocular secretions infected with Chlamydia trachomatis among one another, particularly if they are sharing fomites and cross infection through contaminated fingers [18, 46].

Moreover, the odds of having active trachoma among school-age children who washed their faces without soap were 3.27 times higher compared to those who washed their faces by using soap (AOR = 3.27; 95%CI: 1.19–8.94). This finding is well supported by a systematic review and meta-analysis study [51]. Using soap was the key to abolishing flies, which are the main cause and transmission of trachoma due to the continuous interruption of contact between vectors and children [52].

The odds of having active trachoma among children from households where solid waste was disposed of improperly were 3.02 times higher than those households that disposed of solid waste properly (AOR = 3.02; 95%CI: 1.17, 7.8). This finding is in line with the studies done in the Harari Region [35] and Gazegibela [53]. This may be due to improper solid waste management at the household level, which is a persistent problem in the town that might harbor and support fly breeding [52]. Solid waste is a challenge in Debre Tabor town[54]. Solid waste can create unsanitary conditions to the environment which in turn can lead to pollution of the land and nearby water sources and accelerate outbreaks of vector-borne disease—that is, diseases spread by rodents and insects. This implies that basic sanitation is of paramount importance to prevent trachoma.

In addition, the odds of active trachoma among households having an unimproved type of latrine were 5.18 times higher than those from households who have an improved type of latrine (AOR = 5.18; 95%CI: 1.90–13.69). This finding is parallel with that of Darfur [55], because inadequate sanitation favors the breeding site of flies [18, 56]. As a limitation of the study, school wash facility and livestock factors were not included in the study.

## Conclusion

The prevalence of active trachoma among school-age children was high compared to WHO’s definition of trachoma as a public health problem [57]. Improper home solid waste management, an unimproved type of toilet, an unclean face, the lack of soap during face washing, and a household with more than four family sizes were significantly associated factors to active trachoma. This finding implies the need for mass drug administration to reduce the burden of active trachoma among school age children. Promotion of behavioral determinants through health education programs like keeping facial cleanliness by washing their child’s face with soap, managing solid waste properly, and installing improved latrines to reduce active trachoma needs to be in place. The promotion of the utilization of family planning to improve short birth intervasl, and thereby reduce the number of children in a household would be important.

## Data Availability

The data upon which the result is based could be accessed based on a reasonable request to the corresponding author**.**

## Abbreviations

EPI: Epidemiological Information
IECW: Integrated Eye Care Workers
MDA: Mass Drug Administration
TF: Trichiasis Inflammation Follicular
TI: Trichiasis Inflammation Intensive
WHO: World Health Organization
